# Tissue factor, osteopontin, **α**_v_**β**_3_ integrin expression in microvasculature of gliomas associated with vascular endothelial growth factor expression

**DOI:** 10.1054/bjoc.2000.1150

**Published:** 2000-05-18

**Authors:** S Takano, K Tsuboi, Y Tomono, Y Mitsui, T Nose

**Affiliations:** Department of Neurosurgery, Institute of Clinical Medicine, University of Tsukuba, 1-1-1 Tennoudai, Tsukuba, Ibaraki, 305-8575, Japan; National Institute of Bioscience and Human Technology, Agency of Industrial Science and Technology, Tsukuba, Ibaraki, Japan

**Keywords:** angiogenesis, glioma, osteopontin, tissue factor, vascular endothelial growth factor, α_V_β_3_ integrin

## Abstract

Vascular endothelial growth factor (VEGF) is a potent angiogenic factor in human gliomas. VEGF-induced proteins in endothelial cells, tissue factor (TF), osteopontin (OPN) and α_v_β_3_ integrin have been implicated as important molecules by which VEGF promotes angiogenesis in vivo. Sixty-eight gliomas were immunohistochemically stained with TF, VEGF, OPN and α_v_β_3_ integrin antibody. Twenty-three tumours, six normal brains and nine glioma cell lines were evaluated for their mRNA expression of VEGF and TF by reverse transcription polymerase chain reaction analysis. The data indicated that TF as well as VEGF was a strong regulator of human glioma angiogenesis. First, TF expression in endothelial cells which was observed in 74% of glioblastomas, 54% of anaplastic astrocytomas and none of low-grade astrocytomas, correlated with the microvascular density of the tumours. Double staining for VEGF and TF demonstrated co-localization of these two proteins in the glioblastoma tissues. Second, there was a correlation between TF and VEGF mRNA expression in the glioma tissues. Third, glioma cell conditioned medium containing a large amount of VEGF up-regulated the TF mRNA expression in human umbilical vein endothelial cells. OPN and α_v_β_3_ integrin, were also predominantly observed in the microvasculature of glioblastomas associated with VEGF expression. Microvascular expression of these molecules could be an effective antiangiogenesis target for human gliomas. © 2000 Cancer Research Campaign


British Journal of Cancer (2000) 82(12), 1967–1973
© 2000 Cancer Research Campaign
doi: 10.1054/ bjoc.2000.1150, available online at http://www.idealibrary.com on IDEAL®

### Tissue factor, osteopontin, $\alpha_v\beta_3$ integrin expression in microvasculature of gliomas associated with vascular endothelial growth factor expression

**S Takano[1], K Tsuboi[1], Y Tomono[1], Y Mitsui[2] and T Nose[1]**

[1]Department of Neurosurgery, Institute of Clinical Medicine, University of Tsukuba, 1-1-1 Tennoudai, Tsukuba, Ibaraki 305-8575, Japan; [2]National Institute of Bioscience and Human Technology, Agency of Industrial Science and Technology, Tsukuba, Ibaraki, Japan

**Summary** Vascular endothelial growth factor (VEGF) is a potent angiogenic factor in human gliomas. VEGF-induced proteins in endothelial cells, tissue factor (TF), osteopontin (OPN) and $\alpha_v\beta_3$ integrin have been implicated as important molecules by which VEGF promotes angiogenesis in vivo. Sixty-eight gliomas were immunohistochemically stained with TF, VEGF, OPN and $\alpha_v\beta_3$ integrin antibody. Twenty-three tumours, six normal brains and nine glioma cell lines were evaluated for their mRNA expression of VEGF and TF by reverse transcription polymerase chain reaction analysis. The data indicated that TF as well as VEGF was a strong regulator of human glioma angiogenesis. First, TF expression in endothelial cells which was observed in 74% of glioblastomas, 54% of anaplastic astrocytomas and none of low-grade astrocytomas, correlated with the microvascular density of the tumours. Double staining for VEGF and TF demonstrated co-localization of these two proteins in the glioblastoma tissues. Second, there was a correlation between TF and VEGF mRNA expression in the glioma tissues. Third, glioma cell conditioned medium containing a large amount of VEGF up-regulated the TF mRNA expression in human umbilical vein endothelial cells. OPN and $\alpha_v\beta_3$ integrin, were also predominantly observed in the microvasculature of glioblastomas associated with VEGF expression. Microvascular expression of these molecules could be an effective antiangiogenesis target for human gliomas. © 2000 Cancer Research Campaign

**Keywords**: angiogenesis; glioma; osteopontin; tissue factor; vascular endothelial growth factor; $\alpha_V\beta_3$ integrin

Angiogenesis, the growth of new blood vessels, is a complex process involving the proliferation, migration and protease production of endothelial cells (Folkman and Shing, 1992). Although there are potentially numerous angiogenic factors (Kumar et al, 1998), considerable evidence has accumulated to indicate that vascular endothelial growth factor (VEGF) may be the angiogenic cytokine of central importance in human solid tumours, including human gliomas (Plate et al, 1992; Kim et al, 1993; Millauer et al, 1994). VEGF represents a useful marker and measurable element of glioblastoma angiogenesis (Takano et al, 1996) and is believed to play a bifunctional role in glioblastoma biology leading to both angiogenesis and vasogenic oedema (Dvorak et al, 1995; Zagzag, 1995).

Recently, molecular mechanisms of VEGF-induced angiogenesis have been demonstrated. VEGF stimulates angiogenesis through cooperative mechanisms involving the VEGF-induced proteins in endothelial cells, tissue factor (TF), $\alpha_v\beta_3$ integrin and osteopontin (OPN) (Senger, 1996). For example, VEGF induction of TF in endothelial cells lead to the generation of active thrombin, thus modifying the composition of the extracellular matrix (Senger et al, 1996). VEGF induction of OPN, which is a ligand for the $\alpha_v\beta_3$ integrin, facilitate endothelial cell migration (Senger et al, 1996). Multiple and complex interactions involving VEGF-induced TF, the $\alpha_v\beta_3$ integrin and OPN in endothelial cells are fundamental to the mechanism by which VEGF promotes angiogenesis in vivo (Senger, 1996). In this study, the expression and localization of VEGF-induced molecules, TF, OPN and $\alpha_v\beta_3$

integrin, in endothelial cells were investigated as angiogenesis markers in a series of human gliomas.

*Received 2 June 1999*
*Revised 18 December 1999*
*Accepted 25 January 2000*

*Correspondence to:* S Takano

### MATERIALS AND METHODS

### Tissue preparation

Sixty-eight patients with gliomas (23 glioblastoma, 13 anaplastic astrocytoma, 32 low-grade astrocytoma) were included in the current study. Brain tumour tissues and normal brain tissues (two cases of non-tumour brain and three cases of brain distant far from tumour) were obtained during routine surgical procedures performed for diagnostic or therapeutic indications. A part of the tissues was immediately fixed in 10% phosphate-buffered formalin for 48 h, paraffin-embedded and used for routine pathological diagnosis and immunohistochemistry. Other parts of tissues were immediately frozen with liquid nitrogen and stored at –70°C. Informed consent was obtained from all subjects involved in the current study.

### Antibodies and immunohistochemistry

The Dako LSAB Kit for mouse and rabbit primary antibody (DAKO, Glostrup, Denmark) was used (Takano et al, 1996). Tissue sections were deparaffined and incubated with 10% normal goat serum in phosphate-buffered saline (PBS) for 20 min. The sections were then incubated with a monocolonal anti-tissue factor antibody, #4509 (American Diagnostica Inc., CT, USA) at a dilution of 1/100

(10 μg ml$^{-1}$ IgG), a monoclonal anti-VEGF antibody, MV303 (Toagosei, Tsukuba, Japan), at a dilution of 1/100 (100 μg ml$^{-1}$ IgG), a polyclonal anti-VEGF antibody, A-20 (Santa Cruz Biotech. Inc., CA, USA) at a dilution of 1/100 (1 μg ml$^{-1}$ IgG), a monoclonal osteopontin antibody, MPIIIB10 (1) (Developmental Studies Hybridoma Bank, IA) at a dilution of 1/100 in PBS overnight at 4°C, and a monoclonal anti-human von Willebrand factor antibody (Dako, Glostrup, Denmark) at a dilution of 1/50 (254 μg ml$^{-1}$) in PBS for 60 min at room temperature. Chromatographically purified mouse IgG and rabbit IgG (Dako) at the same IgG concentration were used as negative controls. Sections were incubated with biotin-conjugated goat anti-mouse or anti-rabbit immunogloblin for 10 min, followed by washing in PBS for 10 min. The sections were then incubated with peroxidase-conjugated streptavidin solution for 5 min, followed by washing in PBS for 5 min. Sections were then stained with freshly prepared aminoethylcarbazole solution for 10 min, followed by washing for 5 min in tap water. The sections were then counterstained with haematoxylin and mounted with aqueous mounting media. The intracellular VEGF, TF and OPN immunostaining was assessed separately for tumour and endothelial cells using a semiquantitative scale (–, not detected; + moderate; ++ strong). In another experiment, frozen sections of 18 glioma tissues (seven glioblastoma, four anaplastic astrocytoma, seven low-grade astrocytoma) were stained with a monoclonal $\alpha_V\beta_3$ integrin antibody, 23C6 (Santa Cruz Biotech Inc., CA, USA) at dilution of 1/50 (4 μg ml$^{-1}$ IgG).

In order to investigate the co-localization of different proteins, glioma tissues were double stained. Briefly, tissue sections were incubated with a blocking goat serum following incubation both with a monocolonal TF antibody (1/100 dilution) and a polyclonal VEGF antibody (1/200 dilution) or a monocolonal $\alpha_V\beta_3$ integrin antibody (1/50 dilution) and a polyclonal VEGF antibody (1/200 dilution) or a monoclonal OPN antibody (1/100 dilution) and a polyclonal VEGF antibody (1/200 dilution) for 60 min at room temperature. The sections were incubated with FluoroLink™ Cy™3 labelled goat anti-rabbit IgG (H+L) (Amersham) and FluoroLink™ Cy™2 labelled goat anti-mouse IgG (H+L) (Amersham) at a dilution of 1/1000 for 10 min, and observed using a fluorescence microscope.

### Tumour vascular density

Vascular density was scored using the vasoproliferative component of the MAGS (microscopic angiogenesis grading system) that has been used to quantify angiogenesis in a variety of tumours (Brem et al, 1972; Takano et al, 1996). The number of vessels at 200× field (1.0 mm$^2$) was measured in microvessel 'hot spots' (i.e. microscopic areas containing the most dense collections of microvessels, as initially identified under low-power magnification) with the use of an Olympus microscope, AHBT3 (Olympus, Tokyo, Japan) on von Willebrand factor-stained tissue sections. Vascular density was defined by averaging the number of vessels in the three most vascularized areas.

### Human glioma cell lines and culture conditions

The human glioma cell lines U-251 MG, U-87 MG, and A-172 were obtained from the American Type Culture Collection (Rockville, MD, USA). The human glioma cell lines TK1, TK2, Masu, Mori, Higa were established from glioblastoma at the Department of Neurosurgery, University of Tsukuba. The human glioma cell line, Becker, was a generous gift. Cells were maintained in modified essential medium (MEM) supplemented with 10% fetal calf serum (FCS) in a humidified atmosphere containing 5% carbon dioxide at 37°C.

Human umbilical cord vein endothelial cells (HUVECs) harvested from umbilical cords were a generous gift from Dr Okuda (University of Tsukuba). HUVECs were maintained with collagen coated flasks (Iwaki Glass, Tokyo, Japan) in E300 medium (Kyokuto, Tokyo, Japan) which are designed for HUVEC culture containing 2% FCS, heparin, alpha fibroblast growth factor (aFGF) and epidermal growth factor (EGF).

### RNA isolation and reverse transcription polymerase chain reaction

Total RNA was extracted from 29 frozen tissues (ten glioblastomas, four anaplastic astrocytomas, nine low-grade astrocytomas, six normal brains) and nine glioma cell lines using RNeasy Mini Kit (Qiagen GmbH, Germany) according to the supplier's recommended procedure. Quantitative reverse transcription polymerase chain reaction (RT-PCR) for TF and VEGF mRNA in glioma cells and glioma tissues has been described previously (Potgens et al, 1994). We performed RT-PCR with the GeneAmp™ RNA PCR Kit (Perkin-Elmer Cetus, Norwalk, CT, USA). Briefly, 1 μg of total RNA was reverse transcribed by MuLV reverse transcriptase in the presence of random hexamer, followed by indicated cycles of PCR reaction (95°C for 1 min, 55°C for 1 min, and 72°C for 1 min) in the presence of 2 μM TF specific primers (30 cycles), VEGF specific primers (28 cycles), or the β-actin-specific primers (16 cycles) as a control. The TF primers were designed (Potgens et al, 1994), the reverse primer (5′-CAGTGCAATATAGCATTTGCAGTAGC-3′) is complementary to positions 1005–980, and the forward primer (5′-CTACTGTTTCAGTGTTCAAGCAGTGA-3′) corresponds to positions 723–748 (Scarpati et al, 1987). The VEGF primers (Weindel et al, 1992) included the reverse primer (5′-CCTGGTGAGAGATCTGGTTC-3′) spanning bases 861–842 and the forward primer (5′-TCGGGCCTCCGAAACCATGA-3′) spanning bases 141–160. The β-actin primers (Ng et al, 1985) included the reverse primer (5′-GGAGTTGAAGGTAGTTTCGTG-3′) spanning bases 2429–2409 and the forward primer (5′-CGGGAAATCGTGCGTGACAT-3′) spanning bases 2107–2126. The predicted sizes of the amplified TF and β-actin DNA products were 282 and 214 bp respectively. The VEGF primers were chosen because they amplified exons 3–8 and allowed for distinguishing between the different VEGF splicing variants. PCR products of 516 bp and 648 bp corresponded with $VEGF_{121}$ and $VEGF_{165}$ respectively (Weindel et al, 1992). The quantification of these RT-PCR products levels was performed on a Macintosh computer using the public domain NIH Image program (developed at the US National Institute of Health).

### Glioma-conditioned medium induction of tissue factor mRNA in HUVECs

One times 10$^5$ glioma cells were plated into a 6-well plate. After incubation for 24 h in MEM with 10% FCS, the medium was changed to serum-free MEM. After 48 h incubation, the conditioned medium was harvested and the concentration of VEGF in

 © 2000 Cancer Research Campaign glioma-conditioned medium was measured using Quantikine™ Human VEGF Immunoassay (R&D systems, Minneapolis, MN, USA). Confluent HUVECs in 25-cm$^2$ flasks were cultured in MCDB107 medium with and without VEGF 10 ng ml$^{-1}$ and the above-prepared glioma-conditioned medium (TK2, Becker, Mori, A172) with and without VEGF antibody (MV303, 1/50 dilution) for 18 h. Total RNA was extracted from cultured HUVECs as described earlier, then TF mRNA expression was measured by RT-PCR using TF specific primers.

### Statistical analyses

Vascular density and densitomtric value of VEGF, TF and β-actin were expressed as mean ± standard deviation (s.d.). Statistically significant differences between tumour types were determined using a one-way analysis of variance and the Tukey test. Correlation between VEGF and TF expression was determined by a Pearson correlation matrix with the confidence level determined by Bonferroni probabilities. All *P*-values are two-sided; values are considered statistically significant for $P < 0.05$.

### RESULTS

### Immunolocalization of TF and VEGF in glioma tissues

TF antigen was detected consistently in both the tumour cells and the endothelial cells lining tumour-associated vessels by immunohistochemical staining of formalin-fixed sections of astrocytic tumours. TF antigen was detected in the tumour cells of 21 out of 23 glioblastomas, six of 13 anaplastic astrocytomas, and five of 32 low-grade astrocytomas. TF antigen was detected in the tumour-associated vessels of 17 out of 23 glioblastomas, seven of 13 anaplastic astrocytomas, and none of 32 low grade astrocytomas (Figure 1 and Table 1). VEGF antigen was also detected consistently in both the tumor cells and the endothelial cells lining tumour-associated vessels as described previously (Takano et al, 1996). Immunoreactivity with two VEGF antibodies, MV303 and A20, was very similar. The expression of TF and VEGF in endothelial cells was matched in 54 among 68 cases (+ / +: 21, +/–: 12, –/+: 2, –/–: 33, $\chi^2$ test, $P = 0.0001$). Furthermore, localization of TF and VEGF was very similar, e.g. perinecrotic tumour cells and endothelial cells in the invading edge of the tumour. Moreover double staining with VEGF and TF in glioblastoma tissues demonstrated complete matched distribution of TF and VEGF antigen (Figure 1, E, F). Matched distribution of TF and VEGF was observed in 19 of 23 glioblastomas. In this study, vessel numbers counting factor VIII staining vessels were significantly higher in cases with TF-positive in the endothelial cells ($n = 23$) compared to cases with TF-negative in the endothelial cells ($n = 45$) (mean ± s.d., 60.0 ± 31.7 and 26.9 ± 25.1 respectively. $P < 0.0001$). These results demonstrate the strong linkage between TF and VEGF expression as a marker of angiogenic phenotype of astrocytic tumours.

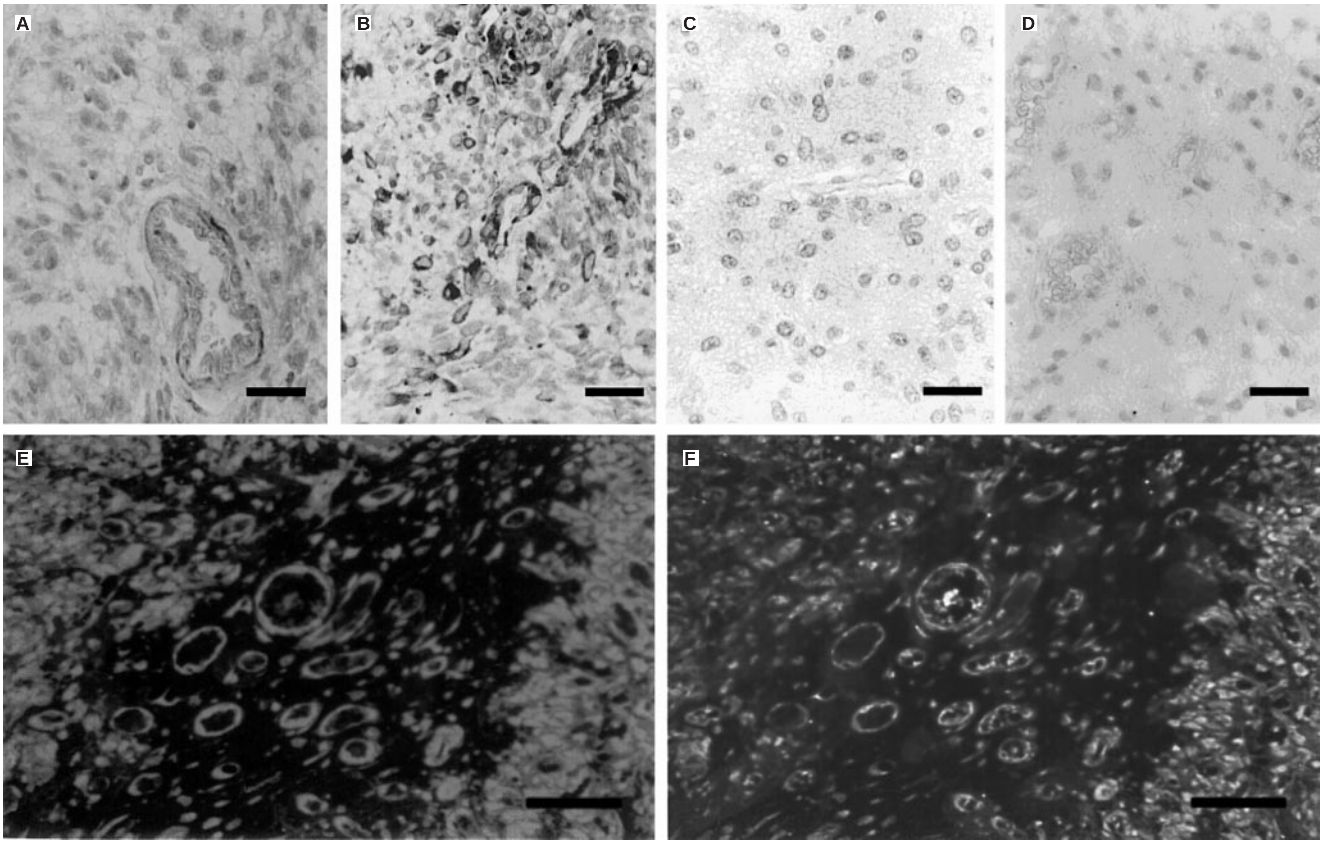

**Figure 1** VEGF and TF immunohistochemistry of human astrocytic tumours. (**A, B**) Glioblastomas and (**C, D**) low-grade astrocytomas (**A, C**) VEGF, (**B, D**) TF staining. In glioblastomas, note the intense immunoreaction for VEGF and TF both in tumour cells and vessel walls. In low-grade astrocytomas, note no immunoreaction for VEGF and TF in either the tumour or vessel walls. Double staining of glioblastoma with VEGF (**E**) and TF (**F**). Note the intense immunoreaction for VEGF and TF both in tumour cells and vessel walls with very similar distribution. Scale bar: 50 μm

**Table 1** Summary of immunohistochemical study in human astrocytic tumours

| | VEGF | | TF | | OPN | | $\alpha_v\beta_3$ integrin | | |
|---|---|---|---|---|---|---|---|---|---|
| | Tumour | EC | Tumour | EC | Tumour | EC | Tumour | EC | Vessel density |
| Glioblastoma | 23/23<br>100% | 18/23<br>78% | 21/23<br>91% | 17/23<br>74% | 20/23<br>87% | 15/23<br>65% | 5/7<br>71% | 7/7<br>100% | 56.7 ± 31.4 |
| Anaplastic astrocytoma | 7/13<br>54% | 7/13<br>54% | 6/13<br>46% | 7/13<br>54% | 11/13<br>85% | 7/13<br>54% | 1/4<br>25% | 1/4<br>25% | 53.0 ± 33.4 |
| Low-grade astrocytoma | 11/32<br>34% | 1/32<br>3% | 5/32<br>16% | 0/32<br>0% | 16/30<br>54% | 7/30<br>23% | 0/7<br>0% | 0/7<br>0% | 18.7 ± 10.7 |

EC: endothelial cell.

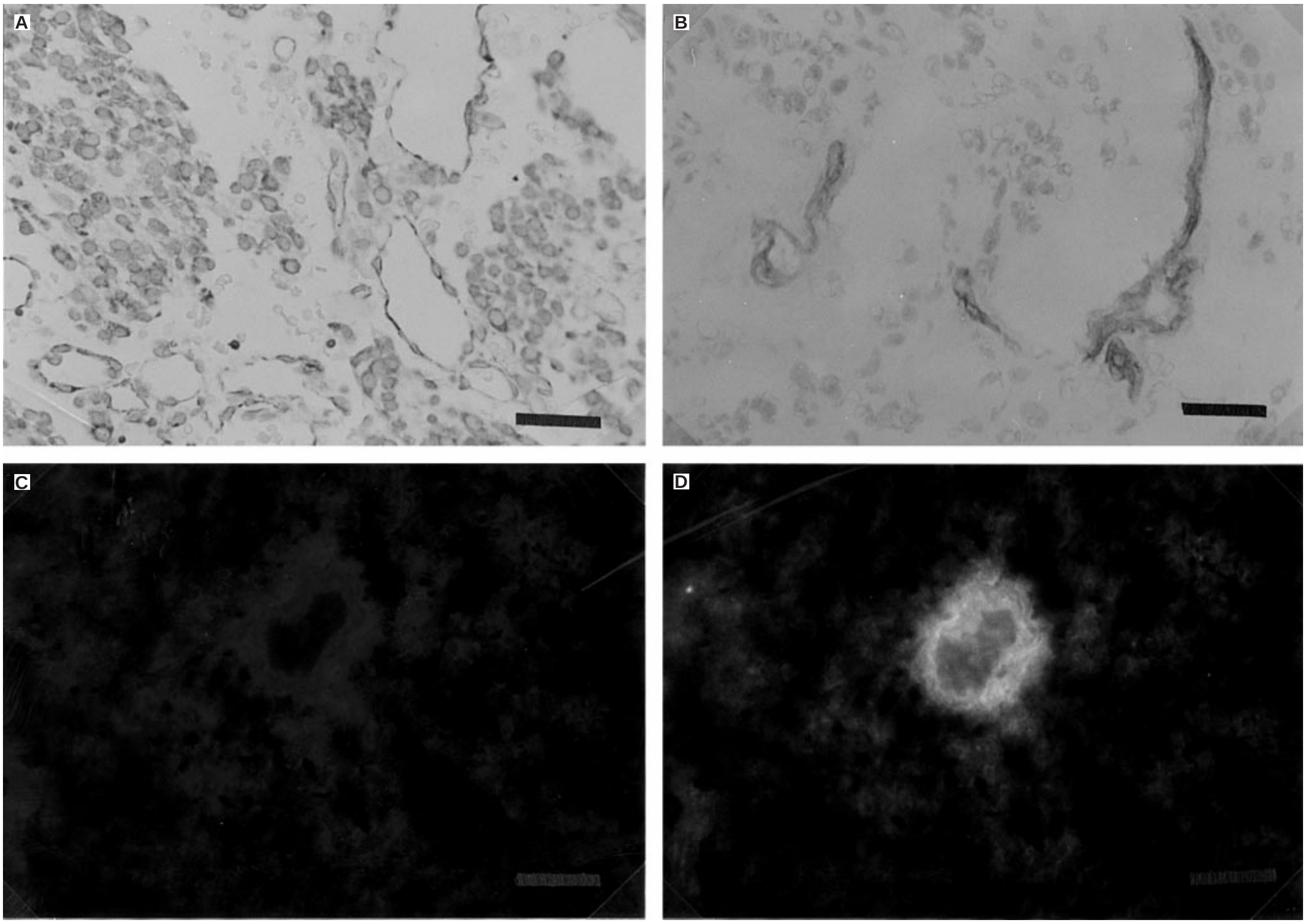

**Figure 2** Immunohistochemistry of glioblastomas. (**A**) Osteopontin; note the intense immunoreaction both in the tumour and vessel walls. (**B**) $\alpha_v\beta_3$ integrin; note the intense immunoreaction in the vessel walls. Double staining with (**C**) VEGF and (**D**) $\alpha_v\beta_3$ integrin; note the same vessel is positive both for VEGF and $\alpha_v\beta_3$ integrin. Scale bar: 50 µm

### Osteopontin and α Vβ3 integrin localization in glioblastoma microvasculature

Osteopontin was localized both in the tumour cells and in the endothelial cells of glioblastomas, with less staining in both the tumour and endothelial cells of anaplastic astrocytomas and low-grade astrocytomas (Figure 2A and Table 1). Double staining with VEGF and OPN antibody demonstrated revealed that VEGF-positive vessels were also positive for OPN (data not shown). Since immunoreactivity with the $\alpha_v\beta_3$ integrin antibody we used was observed only in frozen sections, the number of cases for analysis was limited. $\alpha_v\beta_3$ integrin was localized in the microvasculature of glioblastoma and anaplastic astrocytoma, but not in the microvasculature of low grade astrocytoma and normal brain (Figure 2B). For glioma cells, we observed immunoreactivities with five of seven glioblastomas, one of four anaplastic astrocytomas, but none of seven low-grade astrocytomas. Double staining with VEGF and $\alpha_v\beta_3$ integrin in glioblastoma tissues revealed that VEGF-positive vessels were also positive for $\alpha_v\beta_3$ integrin (Figure 2 C, D).

### TF and VEGF mRNA expression in glioma cells, HUVECs and tissues

RT-PCR was performed to determine whether TF and VEGF would predict the degree of malignancy and angiogenesis in

 *© 2000 Cancer Research Campaign*

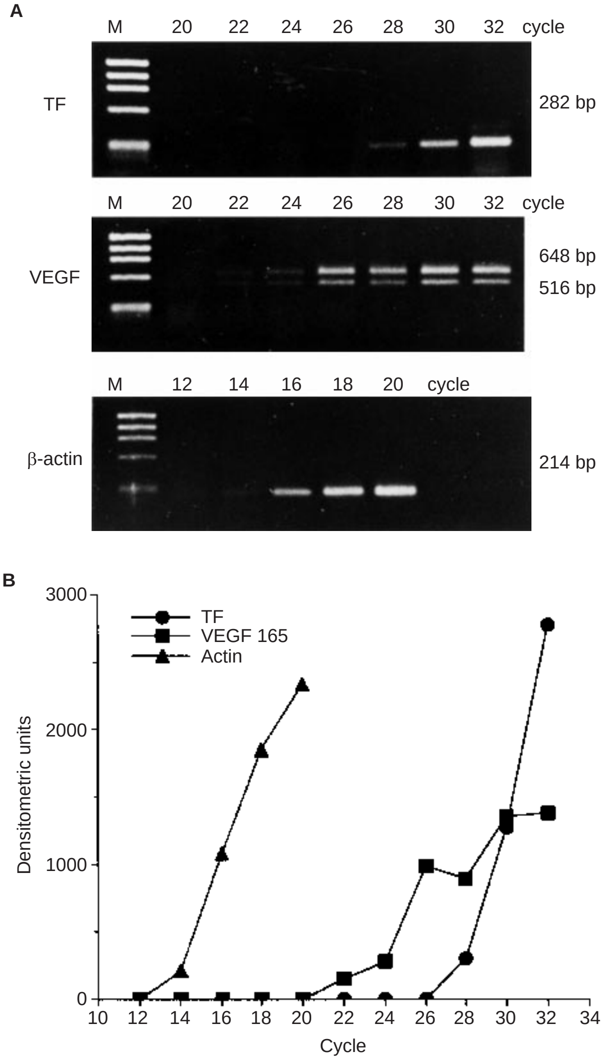

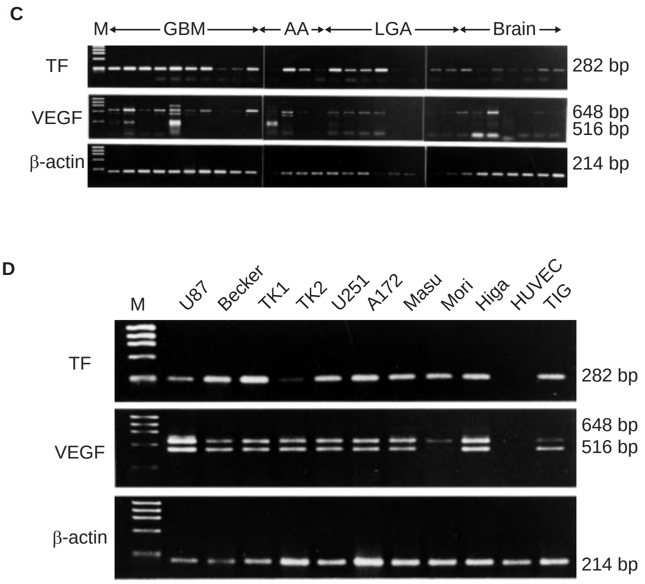

**Figure 3** RT-PCR analysis for the transcripts of VEGF, TF and β-actin in tissues (**A, B, C**) and cell lines (**D**). (**A**) PCR amplification by sequential cycles in the same reaction in one representative glioblastoma tissue. (**B**) Measurement of densitometric units in each cycles in (**A**). The exponential phase of amplification is observed with 30 cycles for TF (●), 28 cycles for VEGF (■) and 16 cycles for β-actin (▲). (**C**) Note both VEGF and TF mRNA expression are observed in astrocytic tumour tissues associating with histological malignancy. (**D**) Note both VEGF and TF mRNA expression is various among nine glioma cell lines (U87, Becker, TK1, TK2, U251, A172, Masu, Mori, Higa), HUVECs and TIG (human dermal fibroblast). Fragments of *øX174/Hae*III were used as markers

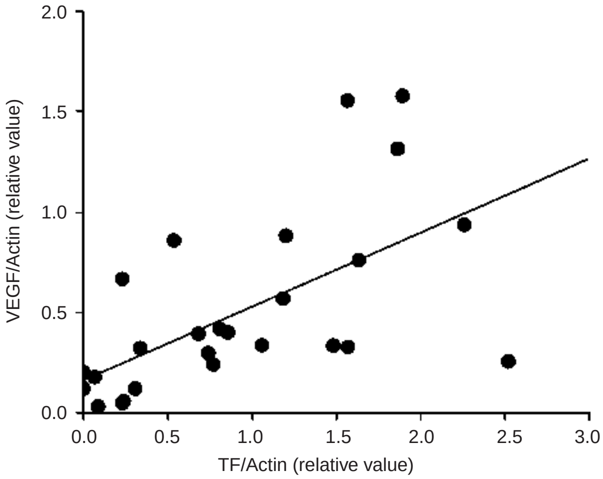

**Figure 4** Correlation between tissue factor and VEGF mRNA expression in glioma tissues evaluated by RT-PCR analysis. There is a significant correlation between TF and VEGF expression ($r = 0.62$, $P < 0.01$)

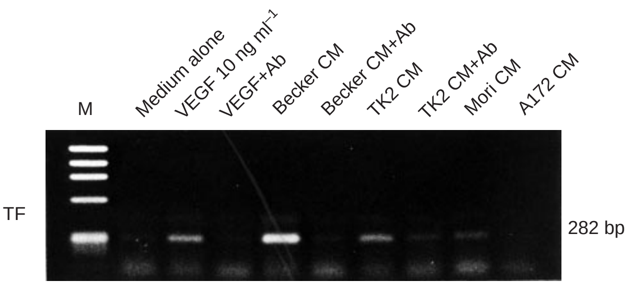

**Figure 5** HUVEC TF mRNA induction with glioma-conditioned medium. TF mRNA expression is not detectable in the control culture of HUVEC (medium alone) and up-regulated with VEGF 10 ng $ml^{-1}$ (VEGF 10 ng $ml^{-1}$). Glioma-conditioned medium contains a large amount of VEGF also up-regulate TF mRNA expression (Becker CM and TK2 CM). VEGF antibody treatment, 1/50 dilution for 18 h abolished the up-regulation of TF induction (Becker CM + Ab, TK2 CM + Ab, VEGF + Ab). The up-regulation of TF mRNA expression with glioma-conditioned medium contains little amount of VEGF is very little (Mori CM and A172 CM)

© 2000 Cancer Research Campaign glioma cells and tissues. For quantitative PCR analysis, we defined the exponential phase of amplification by sequential cycles of PCR. Also in order to compare the relative mRNA of the different transcripts (TF, VEGF and β-actin), amplification was done in the same reaction for each samples (Figure 3A). The appropriate cycles in the exponential phase of amplification for TF, VEGF and β-actin were 30, 28 and 16 cycles respectively (Figure 3B). The 282-bp band derived from TF mRNA was detected in all tissues. RT-PCR analysis by VEGF-specific primers revealed the 648- and 516-bp bands, which represent two molecular forms $VEGF_{165}$ and $VEGF_{121}$ in almost tissues, predominantly 648-bp band. In addition, one additional band larger than 770-bp in two of glioblastomas and one anaplastic astrocytoma was observed. Origin of this band, however, was not determined yet. The results presented in Figure 3C show that both TF and VEGF mRNA levels revealed the degree of malignancy of glioma tissues. When standardized by the amount of β-actin in lane, the relative amounts of TF were 1.36 ± 0.48 in glioblastomas, 0.95 ± 0.90 in anaplastic astrocytomas, 0.90 ± 0.83 in low-grade astrocytomas and 0.17 ± 0.10 in normal brain, and the relative amounts of $VEGF_{165}$ were 1.14 ± 1.03 in glioblastomas, 0.53 ± 0.44 in anaplastic astrocytomas, 0.30 ± 0.11 in low-grade astrocytomas and 0.18 ± 0.25 in normal brain. There was a significant correlation between the TF mRNA and VEGF mRNA levels in glioma tissues (Figure 4, $r = 0.62$, $P < 0.01$). In the nine glioma cell lines the correlation between the TF mRNA and VEGF mRNA levels was weak and not significant (Figure 3D).

VEGF (10 ng $ml^{-1}$) up-regulated the TF mRNA level in HUVECs (Figure 5). Glioma cell (Becker and TK2)-conditioned medium prepared in 48-h serum-free conditions containing a large amount of VEGF (6.3 ng $ml^{-1}$ and 5.1 ng $ml^{-1}$ respectively) measured by enzyme-linked immunosorbent assay (ELISA) up-regulated the TF mRNA in HUVEC at the similar degree with VEGF 10 ng $ml^{-1}$. While this up-regulation was inhibited with the anti-VEGF antibody treatment (MV303, 1/50 dilution) of Becker and TK2 conditioned medium and VEGF (Figure 5). In contrast, up-regulation of TF mRNA was very little with other glioma cell (Mori and A172)-conditioned medium containing little amount of VEGF (0.6 ng $ml^{-1}$ and 0.4 ng $ml^{-1}$ respectively) (Figure 5).

### DISCUSSION

### VEGF and TF play a key role in glioma angiogenesis

Our findings of TF localized to the tumour and endothelial cells and the level of the expression was correlated with the histological malignancy and the microvascular density are of interest particularly in the context of recent data linking TF expression in tumour cells and endothelial cells with angiogenesis (Contrino et al, 1996). TF expression in glioma tissues has been reported (Hamada et al, 1996). However, the role and the mechanism of TF expression in endothelial cells has not yet been determined. TF is closely related to angiogenesis, because TF contributes to the regulation of blood vessel development in early embryogenesis (Carmeliet et al, 1996) and human recombinant tissue factor pathway inhibitor inhibits the growth of cultured HUVECs by inducing apoptosis (Hamuro et al, 1998). TF is also linked to tumour angiogenesis. Expression of TF in the angiogenic endothelial cells may be related to the functional requirements of the sprouting and invading capillary during neovascularization of the tumour (Ott et al, 1998). Tumour growth and angiogenesis were promoted by TF overexpression and inhibited by suppression of TF expression (Zhang et al, 1994). TF expression in endothelial cells appears to be a marker for tumour angiogenesis in gliomas as in breast cancers (Contrino et al, 1996) and non-small-cell lung carcinoma (Koomagi et al, 1998).

A strong link between VEGF and TF expression in solid tumours has been described (Zhang et al, 1994; Koomagi et al, 1998; Ollivier et al, 1998; Shoji et al, 1998). These studies demonstrated the close relationship between tumour TF and tumour VEGF. Our study affords a new insight with regard to the link between VEGF and TF in glioma angiogenesis. First, glioma-conditioned medium containing a large amount of VEGF (Becker- and TK2-conditioned medium) as well as VEGF antigen (10 ng $ml^{-1}$) itself up-regulated TF induction in endothelial cells (Figure 5). The TF up-regulation was neutralized with anti-VEGF antibody treatment of the conditioned medium (Figure 5). This result suggests a strong link between tumour VEGF and endothelial TF that is revealing of in vivo angiogenic conditions. Second, in glioblastoma tissues TF and VEGF were co-localized both in the tumour and the endothelial cells (Figure 1 E,F) and the expression of TF and VEGF mRNA were significantly correlated with each together (Figure 4). Shoji et al (1998) found a strong relationship between the synthesis of TF and VEGF levels in human breast cancer cell lines. In our study, the expression of TF and VEGF in glioma cells in vitro was not significant. VEGF in glioma cells and TF up-regulation in endothelial cells by a paracrine mechanism may, therefore, play a key role in glioma angiogenesis.

### $\alpha_v\beta_3$ integrin, osteopontin and glioma angiogenesis

OPN is expressed at relatively high levels by some tumour cells, including glioma cells (Brown et al, 1994; Saitoh et al, 1995). OPN expression in the tumour microvasculature has not received much attention. OPN was highly expressed in glioblastoma microvasculture co-localizing with VEGF, but less in low-grade astrocytoma and no expression in normal vasculature. OPN mRNA is not detectable in control cultures of endothelial cells. Induction of OPN mRNA in endothelial cells, resulting stimulation of endothelial cell migration has been observed by VEGF 10 ng $ml^{-1}$ (Senger et al, 1996). Induction of OPN expression in endothelial cells is one of the mechanisms by which VEGF promotes angiogenesis. Expression of $\alpha_v\beta_3$ integrin was observed in small vessels of glioblastoma and anaplastic astrocytoma tissues, but not in those of non-neoplastic brain tissues and low-grade astrocytomas, which are consistent with a previous report (Gladson, 1996). We showed co-localization of VEGF and $\alpha_v\beta_3$ integrin in the glioblastoma microvessels using double immunostaining (Figure 3). Time and dose-dependent induction of $\alpha_v\beta_3$ integrin mRNA in endothelial cells by VEGF has been reported (Senger et al, 1996). Induction of $\alpha_v\beta_3$ integrin expression in endothelial cells is likely an important element of the mechanism by which VEGF promotes angiogenesis (Brooks et al, 1994*a*, 1994*b*). Taken together, OPN and $\alpha_v\beta_3$ integrin in the microvasculature of malignant glioma tissues may be induced by VEGF stimulation. Because OPN is an $\alpha_v\beta_3$ ligand, co-induction of OPN and $\alpha_v\beta_3$ in enndothelial cells by VEGF are also one of the markers for glioma angiogenesis.

In summary, the data presented here predict that VEGF and VEGF-induced endothelial molecules TF, $\alpha_v\beta_3$ integrin and OPN

 © 2000 Cancer Research Campaign were well organized in glioma microvasculature and the molecular marker of glioma angiogenesis. There was a strong connection between VEGF and TF expression. VEGF and TF possess several distinct but cooperative mechanisms involving $\alpha_v\beta_3$ integrin and OPN for the regulation of glioma angiogenesis. The cumulative effects of VEGF, TF, $\alpha_v\beta_3$ integrin and OPN on tumour angiogenesis should be viewed as an interactive process rather than as solitary events (Zucker et al, 1998). TF, integrin $\alpha_v\beta_3$ and OPN as well as VEGF expression on small blood vessels in glioblastoma tumours could be an effective target for future therapeutic endeavours in anti-angiogenesis (Takano et al, 1994*a*, 1994*b*, 1994*c*; Gladson, 1996).

### ACKNOWLEDGEMENTS

We gratefully acknowledge Yoshiko Tsukada, Makiko Miyagawa, and Kazuko Morita for the excellent technical assistance; and Takako Yamaguchi for preparation of the manuscript. This study was supported by a Grant-in-Aid for Scientific Research 10671282 to S Takano from Japanese Ministry of Education, Science and Culture.

### REFERENCES

Brem S, Cotran SS and Folkman J (1972) Tumor angiogenesis: a quantitative method for histological grading. *J Natl Cancer Inst* **48**: 347–356

Brooks PC, Clark RAF and Cheresh DA (1994*a*) Requirement of vascular integrin $\alpha_v\beta_3$ for angiogenesis. *Science* **264**: 569–571

Brooks PC, Montgomery AMP, Rosenfeld M, Reisfeld RA, Hu T, Klier G and Cheresh DA (1994*b*) Integrin $\alpha_v\beta_3$ antagonists promote tumor regression by inducing apoptosis of angiogenic vessels. *Cell* **79**: 1157–1164

Brown LF, Papadopoulos-Sergiou A, Berse B, Manseau EJ, Tognazzi K, Perruzzi CA, Dvorak HF and Senger DR (1994) Osteopontin expression and distribution in human carcinomas. *Am J Pathol* **145**: 610–623

Carmeliet P, Macknan N, Mooms L, Luther T, Gressens P, Van Vlaenderen I, Demunck H, Kasper M, Breier G, Evrard P, Muller M, Risau W, Edginton T and Collen D (1996) Role of tissue factor in embryonic blood vessel development. *Nature* **383**: 73–75

Contrino J, Hair G, Kreutzer DL and Ricklcs FR (1996) In situ detection of tissue factor in vascular endothelial cells: correlation with the malignant phenotype of human breast disease. *Nat Med* **2**: 209–215

Dvorak HF, Brown LF, Detmar M and Dvorak AM (1995) Vascular permeability factor/vascular endothelial growth factor, microvascular hyperpermeability, and angiogenesis. *Am J Pathol* **146**: 1029–1039

Folkman J and Shing Y (1992) Mini-review: angiogenesis. *J Biol Chem* **267**: 10931–10934

Gladson CL (1996) Expression of integrin $\alpha_v\beta_3$ in small blood vessels of glioblastoma tumors. *J Neuropathol Exp Neurol* **55**: 1143–1149

Hamada K, Kuratsu J, Saitoh Y, Takeshima H, Nishi T and Ushio Y (1996) Expression of tissue factor correlates with grade of malignancy in human glioma. *Cancer* **77**: 1877–1883

Hamuro T, Kamikubo Y, Nakahara Y, Miyamoto S and Funatsu A (1998) Human recombinant tissue factor pathway inhibitor induces apoptosis in cultured human endothelial cells. *FEBS Lett* **421**: 197–202

Kim KJ, Winer J, Armanini M, Gillett N, Phillips HS and Ferrara N (1993) Inhibition of vascular endothelial growth factor-induced angiogenesis suppresses tumor growth in vivo. *Nature (Lond)* **362**: 841–844

Koomagi R and Volm M (1998) Tissue factor expression in human non-small cell lung carcinoma measured by immunohistochemistry: correlation between tissue factor and angiogenesis. *Int J Cancer* **79**: 19–22

Kumar R, Yoneda J, Bucana C and Fidler IJ (1998) Regulation of distinct steps of angiogenesis by different angiogenic molecules. *Int J Oncol* **12**: 749–757

Millauer B, Schawver LK, Plate KH, Risau W and Ullrich A (1994) Glioblastoma growth inhibited in vivo by a dominant-negative Flk-1 mutant. *Nature (Lond)* **367**: 576–579

Ng SY, Gunning P, Eddy R, Ponte P, Leavitt J, Shows T and Kedes L (1985) Evolution of the functional human β-actin gene and its multipseudogene family: conservation of non-coding regions and chromosomal dispersion of pseudogenes. *Mol Cell Biol* **5**: 2720–2732

Ollivier V, Bentolila S, Chabbat J, Hakim J and de Prost D (1998) Tissue factor-dependent vascular endothelial growth factor production by human fibroblasts in response to activated factor VII. *Blood* **91**: 2698–2703

Ott I, Fischer EG, Miyagi Y, Mueller BM and Ruf W (1998) A role for tissue factor in cell adhesion and migration mediated by interaction with actin-binding protein 280. *J Cell Biol* **140**: 1241–1253

Plate KH, Breier G, Weich HA and Risau W (1992) Vascular endothelial growth factor is a potential tumor angiogenesis factor in human gliomas in vivo. *Nature (Lond)* **359**: 845–888

Potgens AJG, Lubsen NH, van Altena G, Schoenmakers JGG, Ruiter DJ and de Waal RMW (1994) Measurement of tissue factor messenger RNA levels in human endothelial cells by a quantitative RT-PCR assay. *Thromb Haemost* **71**: 208–213

Saitoh Y, Kuratsu J, Takeshima H, Yamamoto S and Ushio Y (1995) Expression of osteopontin in human glioma. Its correlation with the malignancy. *Lab Invest* **72**: 55–63

Scarpati EM, Wen D, Broze GJJ, Miletich JP, Flandermeyer RR, Siegel NR and Sadler JE (1987) Human tissue factor: cDNA sequence and chromosome localization of the gene. *Biochemistry* **26**: 5234–5238

Senger DR (1996) Molecular framework for angiogenesis: a complex web of interactions between extravasated plasma proteins and endothelial cell proteins induced by angiogenic cytokines. *Am J Pathol* **149**: 1–7

Senger DR, Ledbetter SR, Claffey KP, Papadopoulos-Sergiou A, Perruzzi CA and Detmar M (1996) Stimulation of endothelial cell migration by vascular permeability factor/vascular endothelial growth factor through cooperative mechanisms involving the $\alpha_v\beta_3$ integrin, osteopontin, and thrombin. *Am J Pathol* **149**: 293–305

Shoji M, Hancock WW, Abe K, Micko C, Casper KA, Baine RM, Wilcox JN, Danave I, Dillehay DL, Matthews E, Contrino J, Morrissey JH, Gordon S, Edgington TS, Kudryk B, Kreutzer DL and Rickles FR (1998) Activation of coagulation and angiogenesis in cancer – immunohistochemical localization in situ of clotting proteins and vascular endothelial growth factor in human cancer. *Am J Pathol* **152**: 399–411

Takano S, Gately S, Neville ME, Herblin WF, Gross JL, Engelhard H, Perricone M, Eidsvoog K and Brem S (1994*a*) Suramin, an anticancer and angiosuppressive agent, inhibits endothelial cell binding of basic fibroblast growth factor, migration, proliferation, and induction of urokinase-type plasminogen activator. *Cancer Res* **54**: 2654–2660

Takano S, Gately S, Engelhard H, Tsanaclis AMC and Brem S (1994*b*) Suramin inhibits glioma cell proliferation in vitro and in the brain. *J Neuro-Oncol* **21**: 189–201

Takano S, Gately S, Jiang JB and Brem S (1994*c*) A diaminoanthraquinone inhibitor of angiogenesis. *J Pharmacol Exp Ther* **271**: 1027–1033

Takano S, Yoshii Y, Kondo S, Suzuki H, Maruno T, Shirai S and Nose T (1996) Concentration of vascular endothelial growth factor in the serum and tumor tissue of brain tumor patients. *Cancer Res* **56**: 2185–2190

Weindel K, Marme D and Weich HA (1992) AIDS-associated Kaposi's sarcoma cells in culture express vascular endothelial growth factor. *Biochem Biophys Res Commun* **183**: 1167–1174

Zagzag D (1995) Angiogenic growth factors in neural embryogenesis and neoplasia. *Am J Pathol* **146**: 293–309

Zhang Y, Deng Y, Luther T, Muller MM, Zlegler R, Waldnerr R, Stern DM and Nawroth PP (1994) Tissue factor controls the balance of angiogenic and anti-angiogenic properties of tumor cells in mice. *J Clin Invest* **94**: 1320–1327

Zucker S, Mirza H, Conner CE, Lorenz AE, Drews MH, Bahou WR and Jesty J (1998) Vascular endothelial growth factor induces tissue factor and matrix metalloproteinase production in endothelial cells: conversion of prothrombin to thrombin results in progelatinase A activation and cell proliferation. *Int J Cancer* **75**: 780–786

© 2000 Cancer Research Campaign 
